
JOURNAL INFORMATION
==============================
NLM Title Abbreviation: ScientificWorldJournal
Journal ID: ScientificWorldJournal
NLM Journal ID: 101131163
Journal ID: TSWJ

The Scientific World Journal

ISSN: 2356-6140
EISSN: 1537-744X
Publisher: Wiley

ARTICLE INFORMATION
==============================
PMCID: PMC5763770
PMID: 20155234
DOI: 10.1100/tsw.2010.20
Article ID: 256532
Article version: 1
Subjects: Editorial

Editorial: Evaluation of the Project P.A.T.H.S. in Hong Kong: Are the Findings Replicable Across Different Populations?

T.L. Shek Daniel 1 2 3 *
Ma Hing Keung 4
1 Department of Applied Social Sciences, Hong Kong Polytechnic University, Hong Kong
2 Public Policy Research Institute, Hong Kong Polytechnic University, Hong Kong
3 Kiang Wu Nursing College of Macau, Macao
4 Department of Education Studies, Hong Kong Baptist University, Hong Kong
*Daniel T.L. Shek: daniel.shek@polyu.edu.hkAcademic Editor: Joav Merrick

Publication date: 2010
Electronic publication date: 2010 Feb 12
Volume: 10
First page: 178
Last page: 181
Received 2009 Dec 10; Revised 2010 Jan 8; Accepted 2010 Jan 8
Copyright: Copyright © 2010 Daniel Shek and Hing Keung Ma.
Copyright year: 2010
License: This is an open access article distributed under the Creative Commons Attribution License, which permits unrestricted use, distribution, and reproduction in any medium, provided the original work is properly cited.
License URL: https://creativecommons.org/licenses/by/3.0/

Funding: The Hong Kong Jockey Club Charities Trust

==============================
Editorial
Special Issue: Evaluation of the Project P.A.T.H.S.: Replications
*TheScientificWorldJOURNAL* (2010) 10, 178–181
TSW Child Health & Human Development
ISSN 1537-744X; DOI 10.1100/tsw.2010.20

TheScientificWorldJOURNAL
www.thescientificworld.com

### Editorial: Evaluation of the Project P.A.T.H.S. in Hong Kong: Are the Findings Replicable Across Different Populations?

Daniel T.L. Shek[1,2,3,]* and Hing Keung Ma[4]

*[1]Department of Applied Social Sciences, The Hong Kong Polytechnic University; [2]Public Policy Research Institute, The Hong Kong Polytechnic University; [3]Kiang Wu Nursing College of Macau; [4]Department of Education Studies, Hong Kong Baptist University*

**E-mail**: daniel.shek@polyu.edu.hk

*Received December 10, 2009; Revised January 8, 2010; Accepted January 8, 2010; Published February 12, 2010*

---

With reference to the intensification of adolescent developmental problems in Hong Kong[1,2], there are very few systematic and multiyear positive youth development programs in Hong Kong. For the existing youth enhancement programs, they commonly deal with isolated problems and issues in adolescent development (i.e., deficits-oriented programs) and they are relatively short term in nature. To promote holistic development among adolescents in Hong Kong, The Hong Kong Jockey Club Charities Trust initiated and launched a project entitled "P.A.T.H.S. to Adulthood: A Jockey Club Youth Enhancement Scheme". The word "P.A.T.H.S." denotes **P**ositive **A**dolescent **T**raining through **H**olistic **S**ocial Programmes. The Trust invited academics of five universities in Hong Kong to form a research team with The Hong Kong Polytechnic University as the lead institution in order to develop a multiyear universal positive youth development program to promote holistic adolescent development in Hong Kong. Besides developing the program, the research team also provides training for teachers and social workers who implement the program, and carries out longitudinal evaluation of the project[3].

There are two tiers of programs (Tier 1 and Tier 2 programs) in this project. The Tier 1 Program is a universal positive youth development program in which students in Secondary 1 to 3 participate, normally with 20 h of training in the school year at each grade. Because research findings suggest that roughly one-fifth of adolescents will need help of a deeper nature, the Tier 2 Program is generally provided for at least one-fifth of the students who have greater psychosocial needs at each grade (i.e., selective program).

One unique characteristic of the Project P.A.T.H.S. is systematic evaluation of the program. Utilizing the principle of triangulation, various evaluation strategies have been used to evaluate the Tier 1 Program as follows:

1. **Objective Outcome Evaluation**: A randomized group trial with 24 experimental schools and 24 control schools initially has been carried out.
2. **Subjective Outcome Evaluation (Tier 1 Program)**: Both students and program implementers are invited to complete subjective outcome evaluation forms (Form A and Form B, respectively) after completion of the program.
3. **Process Evaluation**: Systematic observations are carried out in randomly selected schools to understand the program implementation details.

*Corresponding author.
©2010 with author.
Published by TheScientificWorld; www.thescientificworld.com

4. **Interim Evaluation**: To understand the process of implementation, interim evaluation is conducted by randomly selecting roughly half of the participating schools in the Experimental and Full Implementation Phases.
5. **Qualitative Evaluation (Focus Groups Based on Students)**: Focus groups involving students based on schools randomly selected from the participating schools are carried out.
6. **Qualitative Evaluation (Focus Groups Based on Program Implementers)**: Focus groups involving instructors based on schools randomly selected from the participating schools are carried out.
7. **Qualitative Evaluation (In-Depth Interviews with Program Implementers)**: Prolonged in-depth interviews with teachers are carried out.
8. **Qualitative Evaluation (Case Study Based on Focus Groups)**: A case study documenting the implementation experience of schools that have incorporated the Tier 1 Program into school formal curriculum is carried out.
9. **Qualitative Evaluation (Student Logs)**: Students are invited to reflect on their experiences after attending P.A.T.H.S. lessons and application of things learned to real life.
10. **Qualitative Evaluation (Student Products)**: Students' weekly diaries are collected after completion of the program. Students' drawings are also collected to reflect on the experiences of the program participants.
11. **Management Information Collected From the Co-Walker Scheme**: Because the co-walkers conducted classroom observations and completed observation forms, such information can give an overall picture about the implementation details in different schools.
12. **Evaluation Based on the Repertory Grid Tests**: Students are randomly selected to complete repertory grid tests that assess their self-identity systems before and after joining the program and perceived changes across years.

Generally speaking, triangulation of the available evaluation findings shows that different stakeholders had positive views about the Tier 1 Program and they perceived the program to be beneficial to the development of the program participants. Most importantly, the findings suggest that the project is effective in promoting positive youth development among Chinese adolescents in Hong Kong. For the Tier 2 Program, subjective outcome evaluation was evaluated by subjective outcome evaluation method. The program participants are invited to complete a subjective outcome evaluation form (Form C) after completion of the program.

To date, evaluation studies of the Project P.A.T.H.S. with reference to the above evaluation strategies have been carried out. These include objective outcome evaluation utilizing a randomized group trial[4]; subjective outcome evaluation based on quantitative and qualitative data collected from the program participants and instructors[5,6]; qualitative evaluation based on focus groups involving students and instructors[7]; in-depth interviews with program implementers, student logs, and student products[8]; process evaluation involving systematic observations of delivery of the program[9]; and interim evaluation[10].

Although the aforementioned mechanisms consistently provide strong evidence that the Project P.A.T.H.S. has a beneficial influence on students[11], there is still the question of whether the evaluation findings can be replicated across populations. Actually, in the realm of science, replication has been regarded as an important strategy for confirming the validity of scientific investigations. Fahs et al.[12] pointed out that "replication of research is essential to the building and continued development of the scientific basis of any discipline.' (p. 67). Reese[13] similarly reminded that "although replication research is often disvalued as 'derivative', it can be an invaluable aid to scientific progress" (p. 1). King[14] also stated that "the most common and scientifically productive method of building on existing research is to replicate an existing finding – to follow the precise path taken by a previous researcher, and then improve the data or methodology in one way or another" (p. 445).

The role of replication in guarding against uncritical acceptance of research findings is also proposed by different researchers. Singh et al.[15] asserted that "replication serves the fundamental role of protecting against the uncritical acceptance of empirical results. It is thus as important as the core academic practices of peer review and publication of research, and is necessary for any stream of scientific inquiry to develop the requisite rigor of a science" (pp. 533–534). Hubbard and Vetter[16] similarly argued that replication "protects against the uncritical assimilation of specious empirical results into the literature. Replications with extensions serve to determine the scope and limits of empirical findings by seeing if they can be generalized to other populations, contexts, time periods, geographical areas, and so on" (p. 153).

Adopting a critical realist position, Tsang and Kwan[17] argued that "although replication cannot yield certain conclusions, it does not follow that it is not desirable to conduct replicated studies or that replication has no epistemic significance. When a replication successfully confirms the findings of the original study, it provides at least some support for the theory concerned. If the two studies are far apart in time, the replication supports the temporal validity of the theory as well. When numerous replications in diverse circumstances are repeatedly successful, it is highly likely that the theory has hit upon some real structure or mechanism in the social world, barring an alternative nonrealist explanation of this success" (p. 759). In short, replication serves an important role in social science research, particularly in the area of program evaluation.

In this special issue, several evaluation papers on the effectiveness of the Tier 1 and Tier 2 Programs, including subjective outcome evaluation based on program participants and implementers, evaluation based on secondary data evaluation, and objective outcome evaluation, are included[18,19,20,21,22,23,24,25]. Generally speaking, the findings arising from these papers are consistent with those reported previously, thus providing evidence for the replication of related research findings. These replicated findings generally suggest that different stakeholders have positive perceptions of the program, workers, and benefits of the program and there is evidence supporting the effectiveness of the program.

### ACKNOWLEDGMENTS

The preparation for this paper and the Project P.A.T.H.S. were financially supported by The Hong Kong Jockey Club Charities Trust.

### REFERENCES

1. Shek, D.T.L. (2007) Tackling adolescent substance abuse in Hong Kong: where we should go and should not go. *TheScientificWorldJOURNAL*: TSW Child Health & Human Development **7**, 2021–2030.
2. Shek, D.T.L. (2006) Adolescent developmental issues in Hong Kong: relevance to positive youth development programs in Hong Kong. *Int. J. Adolesc. Med. Health* **18**, 341–354.
3. Shek, D.T.L. (2006) Conceptual framework underlying the development of a positive youth development program in Hong Kong. *Int. J. Adolesc. Med. Health* **18**, 303–314.
4. Shek, D.T.L., Siu, A.M.H., Lee, T.Y., Cheung, C.K., and Chung, R. (2008) Effectiveness of the Tier 1 Program of Project P.A.T.H.S.: objective outcome evaluation based on a randomized group trial. *TheScientificWorldJOURNAL*: TSW Holistic Health & Medicine **8**, 4–12.
5. Shek, D.T.L. and Ma, H.K. (2007) Subjective outcome evaluation of the Project P.A.T.H.S.: findings based on the perspective of the program participants. *TheScientificWorldJOURNAL* **7**, 47–55.
6. Shek, D.T.L. and Ng, C.S.M. (2009) Subjective outcome evaluation of the Project P.A.T.H.S. (Secondary 2 Program): views of the program participants. *TheScientificWorldJOURNAL*: TSW Child Health & Human Development **9**, 1012–1022.
7. Shek, D.T.L., Sun, R.C.F., and Tang, C.Y.P. (2009) Focus group evaluation from the perspective of program implementers: findings based on the Secondary 2 Program. *TheScientificWorldJOURNAL*: TSW Child Health & Human Development **9**, 992–1002.
8. Shek, D.T.L., Sun, R.C.F., Lam, C.M., Lung, D.W.M., and Lo, S.C. (2008) Evaluations of Project P.A.T.H.S. in Hong Kong: utilization of student weekly diary. *TheScientificWorldJOURNAL*: TSW Holistic Health & Medicine **8**, 13–21.
9. Shek, D.T.L., Sun, R.C.F., and Tang, C.Y.P. (2009) Experimental implementation of the Secondary 3 Program of Project P.A.T.H.S.: observations based on the co-walker scheme. *TheScientificWorldJOURNAL*: TSW Child Health

& Human Development **9**, 1003–1011.

10. Shek, D.T.L., Ma, H.K., and Sun, R.C.F. (2008) Interim evaluation of the Tier 1 Program (Secondary 1 Curriculum) of the Project P.A.T.H.S.: first year of the Full Implementation Phase. *TheScientificWorldJOURNAL*: TSW Holistic Health & Medicine **8**, 47–60.
11. Shek, D.T.L., Ed. (2008) Special issue: evaluation of Project P.A.T.H.S. in Hong Kong. *TheScientificWorldJOURNAL*: TSW Holistic Health & Medicine **8**, 1–94.
12. Fahs, P.S., Morgan, L.L., and Kalman, M. (2003) A call for replication. *J. Nurs. Scholarship* **35(1)**, 67–71.
13. Reese, H.W. (1999) Strategies for replication research exemplified by replications of the Istomina study. *Dev. Rev.* **19**, 1–30.
14. King, G. (1995) Replication, replication. *PS: Polit. Sci. Politics* **28(3)**, 444–452.
15 Singh, K., Ang, S.H., and Leong, S.M. (2003). Increasing replication for knowledge accumulation in strategy research. *J. Management* **29**, 533–549.
16. Hubbard, R. and Vetter, D.E. (1996) An empirical comparison of published replication research in accounting, economics, finance, management, and marketing. *J. Bus. Res.* **35,** 153–164.
17. Tsang, E.W.K. and Kwan, K.M. (1999) Replication and theory development in organizational science: a critical realist perspective. *Acad. Manage. Rev.* **24(4)**, 759–780.
18. Shek, D.T.L. (2010) Objective outcome evaluation of the Project P.A.T.H.S. in Hong Kong: findings based on individual growth curve models. *TheScientificWorldJOURNAL:* TSW Child Health & Human Development **10**, 182–191. DOI 10.1100/tsw.2010.18.
19. Ma, H.K. and Shek, D.T.L. (2010) Subjective outcome evaluation of a positive youth development program in Hong Kong: profiles and correlates. *TheScientificWorldJOURNAL:* TSW Child Health & Human Development **10**, 192–200. DOI 10.1100/tsw.2010.2.
20. Tsang, S.K.M., Hui, E.K.P., Shek, D.T.L., and Law, B.C.M. (2010) Subjective outcome evaluation of the Project P.A.T.H.S.: findings based on the perspective of the program implementers (Secondary 1 Program). *TheScientificWorldJOURNAL:* TSW Child Health & Human Development **10**, 201–210. DOI 10.1100/tsw.2010.22.
21. Shek, D.T.L. and Yu, L. (2010) Subjective outcome evaluation of the Project P.A.T.H.S.: descriptive profiles and correlates. *TheScientificWorldJOURNAL:* TSW Child Health & Human Development **10**, 211–223. DOI 10.1100/tsw.2010.23.
22. Shek, D.T.L. and Sun, R.C.F. (2010) Subjective outcome evaluation based on secondary data analyses: the Project P.A.T.H.S. in Hong Kong. *TheScientificWorldJOURNAL:* TSW Child Health & Human Development **10**, 224–237. DOI 10.1100/tsw.2010.4.
23. Siu, A.M.H. and Shek, D.T.L. (2010) Secondary data analyses of conclusions drawn by the program implementers of a positive youth development program in Hong Kong. *TheScientificWorldJOURNAL:* TSW Child Health & Human Development **10**, 238–249. DOI 10.1100/tsw.2010.1.
24. Shek, D.T.L. and Ma, C.M.S. (2010) Subjective outcome evaluation findings: factors related to the perceived effectiveness of the Tier 2 Program of the Project P.A.T.H.S. *TheScientificWorldJOURNAL:* TSW Child Health & Human Development **10**, 250–260. DOI 10.1100/tsw.2010.19.
25. Lee, T.Y. and Shek, D.T.L. (2010) Positive youth development programs targeting students with greater psychosocial needs: a replication. *TheScientificWorldJOURNAL:* TSW Child Health & Human Development **10**, 261–272. DOI 10.1100/tsw.2010.3.

**This article should be cited as follows:**

Shek, D.T.L. and Ma, H.K. (2010) Editorial: evaluation of the Project P.A.T.H.S. in Hong Kong: are the findings replicable across different populations? *TheScientificWorldJOURNAL:* TSW Child Health & Human Development **10**, 178–181. DOI 10.1100/tsw.2010.20.
